# Examining the Relationship Between Grit and Foreign Language Performance: Enjoyment and Anxiety as Mediators

**DOI:** 10.3389/fpsyg.2021.666892

**Published:** 2021-06-24

**Authors:** Eerdemutu Liu, Junju Wang

**Affiliations:** School of Foreign Language and Literature, Shandong University, Jinan, China

**Keywords:** grit, foreign language enjoyment, foreign language anxiety, foreign language performance, second language learning

## Abstract

The relationship between grit and success has been investigated extensively in various contexts. However, the association between grit and language performance, especially in a Chinese high school context, remains underexplored. This study investigates grit, the positive emotion of enjoyment, the negative emotion of anxiety, foreign language performance, and how enjoyment and anxiety mediate the relationship between grit and foreign language performance. A questionnaire was administered to 697 Chinese high school students, followed by a language test after 2 weeks. The results showed that more than half of the students had a moderate-high level of grit and foreign language enjoyment and that nearly half of them experienced a low-moderate level of foreign language anxiety. It was also found that grit, foreign language enjoyment, and foreign language performance were positively correlated with each other, and all three variables were negatively correlated with anxiety. Both foreign language enjoyment and foreign language anxiety mediated the relationship between grit and foreign language performance to a significant degree, and the mediating effect of foreign language anxiety was stronger than that of foreign language enjoyment.

## Introduction

For decades, the means by which cognitive and non-cognitive factors influence language learning outcomes has been of interest to many second language researchers. Grit, as a non-cognitive individual trait, plays a crucial role in personal achievement (Duckworth et al., 2007; Duckworth and Quinn, 2009) and psychological constructs such as depression and motivation (Steinmayr et al., 2018; Datu et al., 2019). However, few studies have explored the relationship between grit and language learning performance, despite grit's prominent role in language learning compared to other subjects (MacIntyre et al., 2019b).

Foreign language enjoyment and foreign language anxiety are the most common emotions experienced by second language learners in the learning process (Dewaele and MacIntyre, 2014). Extensive research has confirmed the key roles of these two distinct emotions in language learning performance (Dewey et al., 2018; Li, 2020). However, how these two emotions interact with grit and mediate the role of grit in language learning has been underexplored. This study, therefore, aims to explore the relationships between grit, foreign language enjoyment, foreign language anxiety, foreign language performance, and how enjoyment and anxiety mediate the relationship between grit and foreign language performance.

## Literature Review

### Grit and Academic Success

Grit is a non-cognitive and stable higher-order personality trait encompassing two lower-order components: consistency of interest and perseverance of effort. Differential levels of grit can lead to a range of outcomes for individuals with similar cognitive abilities (Duckworth et al., 2007). What distinguishes grit from other conceptually relevant constructs, such as conscientiousness, is its delineation of a person's long-term commitment to a goal despite challenges and adversity (Duckworth and Quinn, 2009).

An extensive body of research has revealed that grit can predict academic success, but the findings of this research have been inconsistent. Whereas, grit has been found to be positively correlated with undergraduate students' self-reported grades (Strayhorn, 2014) and positively correlated with the doctoral students' academic success (Cross, 2014), it was not a significant predictor when controlling for previous scores (Chang, 2014) or when controlling for other personality traits (Ivcevic and Brackett, 2014).

In the field of second language acquisition, grit is considered to play an even greater role (MacIntyre et al., 2019b) and is thought to be a key factor in language learning (Keegan, 2017). For example, Kramer et al. (2018) discovered relationships between both grit and vocabulary scores and grit and extensive reading times. A study conducted by Wei et al. (2019) indicated a link between grit and foreign language performance. Likewise, Robins (2019) proved that grit and perseverance of effort, one of its component facets, were correlated with both students' GPA and their retention in an online context.

### Grit and Emotions

Research findings have consistently indicated a significant correlation between grit and emotions (Singh and Jha, 2008; Hill et al., 2016; Datu et al., 2019). For example, gritty individuals were found to be more optimistic (Duckworth and Duckworth, 2016), less discouraged when faced with adversity (Duckworth and Eskreis-Winkler, 2013), and less likely to experience negative emotions (Datu et al., 2019). Studies have confirmed that grit is positively correlated with passion (Sigmundsson et al., 2020), resilience (Calo et al., 2019; Shakir et al., 2020), well-being (Moen and Olsen, 2020), motivation (Steinmayr et al., 2018; Karlen et al., 2019), positive affect (Singh and Jha, 2008; Hill et al., 2016), happiness, and life satisfaction (Singh and Jha, 2008). Grit is negatively correlated with burnout (Moen and Olsen, 2020; Shakir et al., 2020), negative affect (Singh and Jha, 2008), depression (Datu et al., 2019), and perceived stress (Moen and Olsen, 2020).

In a study by Changlek and Palanukulwong (2015), it was found that grit was positively correlated with motivation and negatively correlated with anxiety. Similarly, Datu et al. (2018) found that grit positively predicted positive affect, life satisfaction, and interdependent happiness and negatively predicted psychological distress. Lee (2020) found that the aspect of grit entailing perseverance of effort, rather than that of consistency of effort, predicted L2 willingness to communicate.

### Emotions and Foreign Language Performance

Emotions play an important role in foreign language learning (MacIntyre et al., 2019b). To a large extent, the absence of emotions signifies boredom and/or the absence of the mind (Dewaele and Pavelescu, 2019).

Among the negative emotions, anxiety has been the most widely studied in the literature on second language acquisition (Horwitz, 2010; MacIntyre and Gregersen, 2012a; MacIntyre, 2017) and has been regarded as one of the emotions with the greatest influence on foreign language outcomes (Dewaele and MacIntyre, 2014). Despite mixed results throughout the literature, it has generally been found to be negatively correlated with second language achievement in various contexts (Phillips, 1992; Aida, 1994; Dewey et al., 2018).

Of the positive emotions, foreign language enjoyment has received the greatest amount of attention from researchers (Dewaele and Li, 2020). Research findings have consistently revealed that foreign language enjoyment is related to second language achievement in different cultural contexts (Dewaele and Alfawzan, 2018; Li, 2020).

As for the relationship between enjoyment and anxiety, studies have consistently found these to be the two most common emotions among foreign language learners (Dewaele and MacIntyre, 2014; Dewaele and Li, 2020); the two emotions are related (Dewaele et al., 2018; Li et al., 2019). However, they are two distinct emotions rather than “opposite ends of a dimension” (Dewaele and MacIntyre, 2014, p. 261), which indicates that an increase in one does not necessarily lead to a decrease in the other.

Several studies have investigated the relationship between these two emotions and foreign language performance, but they have reported inconsistent findings. Dewaele and Alfawzan (2018), for example, found that enjoyment has a stronger correlation with foreign language performance than with anxiety. Dewey et al. (2018) reported that anxiety was the best predictor of students' oral proficiency, whereas enjoyment was not a significant predictor. In a sample of Chinese middle school students, Wei et al. (2019) found that enjoyment played a mediating role between grit and foreign language performance. Similarly, a study conducted by Li et al. (2019) indicated a general tendency for anxiety to be a stronger predictor of self-perceived English proficiency and actual scores than enjoyment.

## Research Questions

Despite an abundance of studies and the impact of their findings, the relationships among grit, emotions, and foreign language learning outcomes have been underexplored, particularly in Chinese high school students. Thus far, the possible mediating roles that emotions, both positive and negative, play in this dynamic remain largely unknown. Given these gaps in the research, the present study aims to explore these relationships and examine the mediating effect of emotions on the relationship between grit and foreign language performance (hereafter FLP). Specifically, it focuses on the two emotions of foreign language enjoyment (hereafter FLE) and foreign language anxiety (hereafter FLA) and addresses the following research questions:

RQ1: What are the general profiles of students' grit, FLE, and FLA?
RQ2: What are the relationships among grit, FLE, FLA, and FLP?
RQ3: Do FLE and FLA mediate the relationship between grit and FLP?

## Methods

### Participants

A total of 697 students from three senior high schools in Northern China participated in this study (including one regular high school, one district key high school, and one city key high school). There were 385 boys and 312 girls, with an age range from 14 to 16 years (*M* = 15.62; *SD* = 0.58). All the students were Chinese L1 users, and English was their only foreign language. None of them had had overseas experiences, and their English learning had followed the same curriculum regulated by the Ministry of Education of China. Every week, they had six English classes, each lasting 40 min. Each semester, they took at least two English tests, which conformed to the requirements of the National College Entrance Examination. When the study was conducted, these students were in their first year of study and had a lower intermediate level of English proficiency. Detailed information on the students is shown in Table 1.

**Table 1:** Demographic information of the participants.

**School**	**Size**.	**Boys**	**Girls**	**Mean age (SD)**
A	193	123	70	15.57 (0.57)
B	325	167	158	15.61 (0.57)
C	179	95	84	15.69 (0.62)
Tot	697	385	312	15.62 (0.58)

### Instruments

#### Grit Scale

An 8-item grit scale was used to assess students' grit levels. This scale consisted of two dimensions, namely, the persistence of effort and consistency of interest (see Duckworth and Quinn, 2009). Sample items included “I am a hard worker” and “I cannot persist with a task that takes more than 1 month.” The items were rated on a 5-point Likert scale ranging from 1 (strongly disagree) to 5 (strongly agree). Four negatively phrased items were reverse-coded so that higher scores reflected higher levels of grit for all items.

The scale demonstrated good internal reliability among Chinese adolescents (Li J. et al., 2018; Wei et al., 2019; Feng and Lan, 2020). In this study (*N* = 697), the Cronbach's alphas of the global grit scale and its two subscales of persistence of effort and consistency of interest were 0.73, 0.71, and 0.69, respectively.

#### Chinese Version of Foreign Language Enjoyment Scale

The 11-item FLE scale (Li C. et al., 2018) used to assess students' levels of enjoyment in the Chinese language learning context is a modification of the original FLE scale (Dewaele and MacIntyre, 2014). It consists of three dimensions, namely, FLE-Private, FLE-Teacher, and FLE-Atmosphere. Sample items included “I enjoy learning English,” “The teacher is friendly,” and “We form a tight group.” Students' responses were recorded on a 5-point Likert scale, ranging from 1 (strongly disagree) to 5 (strongly agree), with all items positively phrased.

The scale demonstrated good internal consistency in previous studies in the Chinese context (Wei et al., 2019; Li, 2020). In this study (*N* = 697), the Cronbach's alphas of global FLE scale and its three subscales FLE-Private, FLE-Teacher, and FLE-Atmosphere were 0.848, 0.75, 0.72, and 0.76, respectively.

#### Foreign Language Anxiety Scale

The 33-item FLA scale (Horwitz et al., 1986) was adopted in this study to assess students' anxiety in a classroom language learning context. Items were measured on a 5-point Likert scale ranging from 1 (strongly disagree) to 5 (strongly agree). Sample items included “I tremble when I know that I'm going to be called on in language class” and “The more I study for a language test, the more confused I get.” Five negatively phrased items in this scale were reverse-coded so that higher scores indicated higher levels of anxiety for all items.

Previous studies have demonstrated the scale's good internal consistency in the Chinese context (Wang, 2003; Shao et al., 2013; Li and Xu, 2019). In this study (*N* = 697), the Cronbach's alpha was 0.949.

#### English Achievement Test

The English test used to assess students' FLP was developed by a team of English teachers. It was designed to be completed within 120 min, with a maximum score of 150 points. Following the example of the National College Entrance Examination, a prominent standardized test in China, the test consisted of six parts: listening comprehension, reading comprehension, a multiple-choice cloze test, fill-in-the-blanks, error correction, and essay writing. The first three parts were objective items, and the remaining three parts were subjective items. These were anonymously scored once each by two teachers. The inter-rater reliability was 0.91, above the threshold of good reliability (0.80) (McHugh, 2012). The students' final scores were determined by averaging the scores given by the two teachers.

Statistical analysis indicated that the test was reliable and valid for assessing students' FLP. The coefficients of item difficulty were obtained by dividing the number of students who answered correctly by the total number of students. The range of item difficulty was 0.42–0.57, within the average difficulty range of 0.25–0.75 (Sim and Rasiah, 2006). The discrimination indices were calculated by subtracting the percentage of the successes of the low achievers (bottom 27%) from those of the high achievers (top 27%). The range of discrimination values obtained was 0.32–0.42, which were above the threshold of 0.3 (Ebel and Frisbie, 1986).

### Procedure and Data Analysis

Before the survey was conducted, we first obtained consent from the students' headmasters, class teachers, and English teachers. As most of the participants were teenagers, we also sent emails or text messages to the students' parents for their consent. When administering the questionnaire and the test, we informed the students of the research objectives and ensured the confidential treatment of their responses. All of the students were willing to participate in the study.

First, the composite questionnaire was administered to the participants in three high schools in a paper-and-pen format in November 2020. The students completed the questionnaires during class time. Two weeks later, they took the English test. The test papers and answer sheets were collected for later scoring and analysis once the test time had elapsed. Of the 716 questionnaires collected, 697 were valid. The proportion of valid responses was 97.3%.

We then analyzed the missing data and checked for the normality distribution. The missing data constituted <1% of all possible responses. The result of Little's (1988) missing completely at random (MCAR) test did not indicate any pattern (Schafer and Graham, 2002). We also used the multiple imputation method to impute the missing data (Larson-Hall, 2015). As Table 2 shows, the Z-standardized values of skewness and kurtosis of all four variables (grit, FLE, FLA, and FLP) were considerably lower than 2.58, the suggested threshold of normality in a sample larger than 200 (Ghasemi and Zahediasl, 2012).

**Table 2:** Means, standard deviations, Z-standardized values of skewness and kurtosis of the key variables.

	***M***	***SD***	***Zs_***kewness***_***	***Z_***Kurtosis***_***
Grit	26.76	5.00	−0.21	0.06
FLE	40.85	7.01	−0.22	−0.16
FLA	83.67	23.84	0.20	−0.46
FLP	104.36	20.84	−0.78	0.15

*FLE, Foreign Language Enjoyment; FLA, Foreign Language Anxiety; FLP, Foreign Language Performance*.

To interpret the effect size of correlation, we followed Plonsky and Oswald's (2014) guidelines and regarded 0.25 as a small effect size, 0.40 as a medium effect size, and 0.60 as a large effect size. To best capture the patterns using these different scales, we subdivided the scores into four levels based on previous studies (Liu and Jackson, 2008; Shao et al., 2013; Li, 2020). Within the grit score range of 8–40, a score of 8–15 was identified as a low level, 16–23 as a low-moderate level, 24–31 as a moderate-high level, and 32–40 as a high level. Within the enjoyment score range of 11–55, a score of 11–21 was identified as a low level, 22–32 as a low-moderate level, 33–43 as a moderate-high level, and 44–55 as a high level. Within the anxiety score range of 33–165, a score of 33–65 was identified as a low level, 66–98 as a low-moderate level, 99–131 as a moderate-high level, and 132–165 as a high level.

Finally, we conducted a statistical analysis using SPSS 25. Descriptive statistics were used to determine the frequencies, means, and standard deviations of grit, FLE, and FLA. Pearson's correlation analysis was used to define the relationships between grit, FLE, FLA, and FLP. Parallel mediation analysis was conducted using Process v3.2 (Model 4) in SPSS 25 to explore the mediating effect of FLE and FLA between grit and FLP.

## Results

### General Profiles of Students' Grit, FLE, and FLA

We found that more than half of the students had a moderate-high level of both grit and enjoyment, while nearly half of them were at a low-moderate level of anxiety.

As Table 3 shows, 20 (2.9%) of the students demonstrated a low level of grit, 203 (29.1%) a low-moderate level, 388 (55.7%) a moderate-high level, and 86 (12.3%) a high level. The analysis revealed that 3 (0.4%) of the students showed low FLE, 97 (13.9%) a low-moderate level, 380 (54.5%) a moderate-high level, and 217 (31.1%) a high level. The findings also show that 177 (25.4%) of the students reported a low level of anxiety, 338 (48.5%) a low-moderate level, 165 (23.7%) a moderate-high level, and 17 (2.4%) a high level.

**Table 3:** The detailed information of grit, FLE, and FLA levels.

**Variables**	**Levels and sizes**			
	**Low**	**Low-moderate**	**Moderate-high**	**High**
Grit	20	203	388	86
FLE	3	97	380	217
FLA	177	338	165	17

*FLE, Foreign Language Enjoyment; FLA, Foreign Language Anxiety; FLP, Foreign Language Performance*.

**Figure 1:**
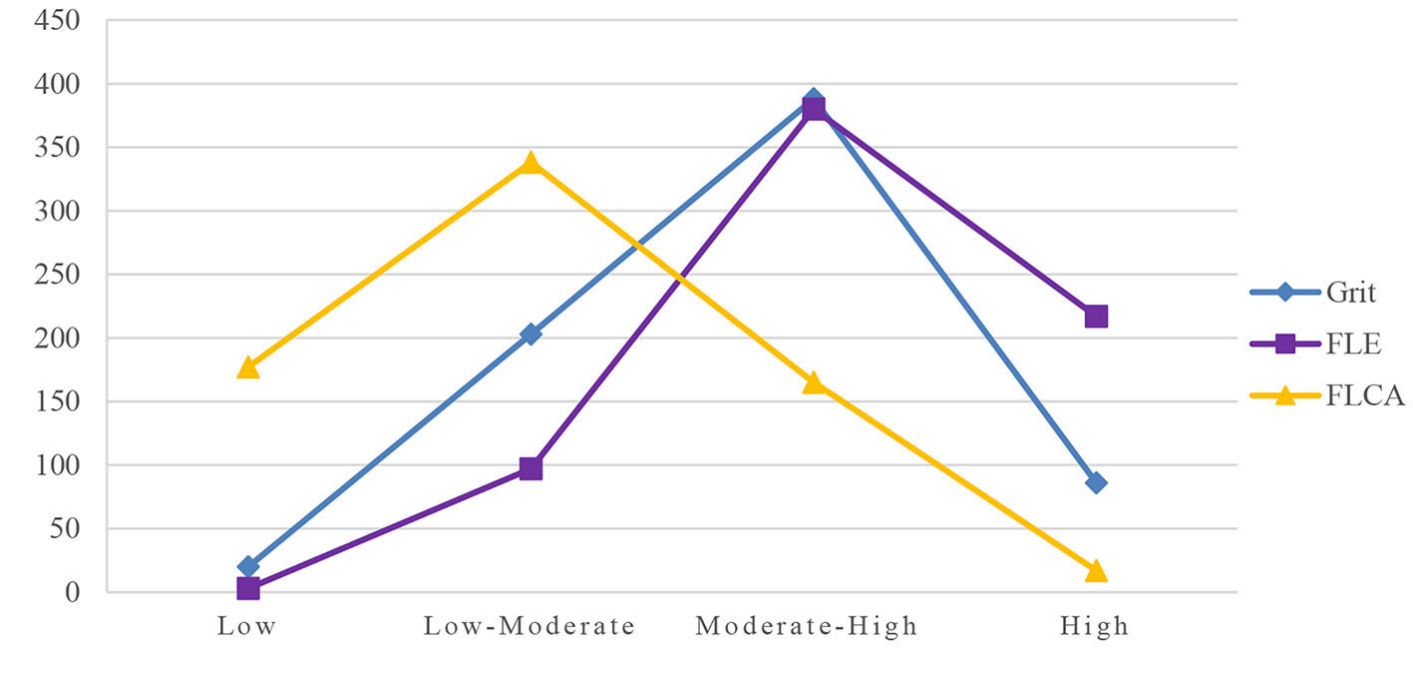
The tendencies of Grit, FLE, and FLA levels.

Figure 1 shows that students' grit levels and enjoyment levels followed similar general tendencies. The number of students who reported a moderate-high level of anxiety was much smaller than the number of those who reported a low-moderate level. This means that the students in this study had a generally healthy emotional state and a favorable level of grit. In general, they were more possessed of the facilitating forces of grit and enjoyment than the inhibitions of anxiety.

In addition, the similar patterns of grit level and enjoyment level may also suggest that grit and enjoyment are two convergent factors that strengthen each other. In contrast, anxiety is in divergent correlation with grit and enjoyment. In other words, a high level of grit and enjoyment in students ensures a lower level of anxiety and vice versa.

### Correlations Between Grit, FLE, FLA, and FLP

The correlation analysis revealed that all four variables of interest were significantly correlated with each other (Table 4).

**Table 4:** Pearson's correlation analysis among key variables.

**Variables**	**1**	**2**	**3**	**4**
Grit	1			
FLE	0.44^**^	1		
FLA	−0.43^**^	−0.50^**^	1	
FLP	0.13^**^	0.27^**^	−0.35^**^	1

*FLE, Foreign Language Enjoyment; FLA, Foreign Language Anxiety; FLP, Foreign Language Performance*;

** *p < 0.01*.

We found that students' grit was significantly correlated with both classroom emotions and FLP. More specifically, it was positively correlated with enjoyment (*r* = 0.44, *p* < 0.01) and negatively correlated with anxiety (*r* = −0.43, *p* < 0.01), with medium effect sizes. Grit was also found to be positively correlated with FLP (*r* = 0.13, *p* < 0.01) with a small effect size. The results indicate that students with a higher level of grit tend to score higher in FLP. In general, the students with a higher level of grit tended to perform better on the test and experienced a higher level of enjoyment and a lower level of anxiety.

FLA was found to be significantly correlated with FLE (*r* = −0.50, *p* < 0.01), with an effect size between medium and large, and negatively correlated with FLP (*r* = −0.35, *p* < 0.01) with an effect size between small and medium. Given FLA's negative correlation with grit (*r* = −0.43, *p* < 0.01), these findings suggest that the students with lower levels of anxiety tended to experience higher levels of enjoyment and higher scores on the test.

FLE was found to be positively correlated with FLP (*r* = 0.27, *p* < 0.01) with an effect size between small and medium. Given that FLE was correlated positively with grit (*r* = 0.44, *p* < 0.01) and negatively with FLA (*r* = −0.50, *p* < 0.01), the results suggest that the students who experienced more enjoyment tended to have higher levels of grit and less anxiety regarding foreign language learning.

Finally, FLP was found to be positively correlated with grit (*r* = 0.13, *p* < 0.01) and FLE (*r* = 0.27, *p* < 0.01) and negatively correlated with FLA (*r* = −0.35, *p* < 0.01). The results revealed that the students with higher test scores tended to have higher grit and FLE, but less FLA.

### Mediating Effect of FLE and FLA on Grit and FLP

**Figure 2:**
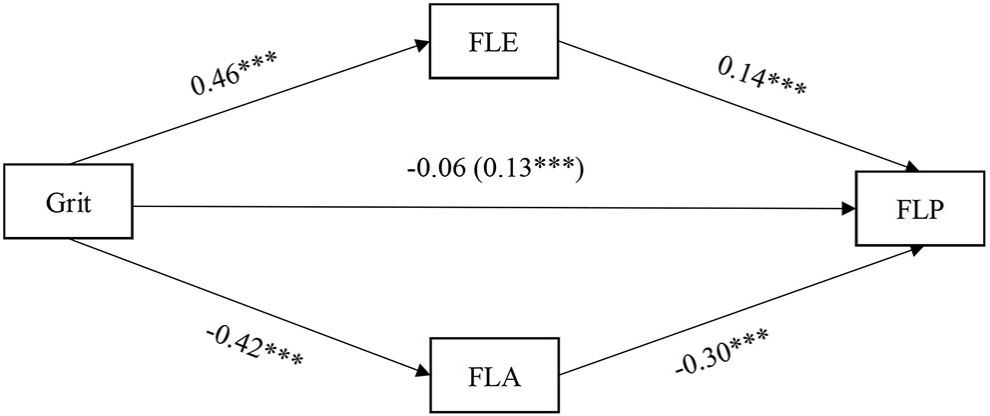
The model of multiple mediation analysis. Values on paths are path coefficients (standardized βs). Path coefficient in parentheses is total effect. FLE, Foreign Language Enjoyment; FLA, Foreign Language Anxiety; FLP, Foreign Language Performance.

To test whether FLA and FLE mediate the relationship between grit and FLP, multiple mediation analysis was conducted using PROCESS v3.2 Statistical Model 4, with gender and age as covariates (Figure 2). All of the variables used in this model were mean-centered to minimize multicollinearity.

As illustrated in Table 5, grit was found to be significantly related to FLP when the two mediators were ignored (β = 0.13, *t* = 3.39, *p* < 0.001). Grit was significantly associated with FLE (β = 0.46, *t* = 32.20, *p* < 0.001) and FLA (β = −0.42, *t* = −29.40, *p* < 0.001). FLE (β = 0.14, *t* = 7.96, *p* < 0.001) and FLA (β = −0.30, *t* = −16.92, *p* < 0.001) were significantly related to FLP when grit was controlled. Furthermore, grit was not found to be significantly related to FLP (β = −0.06, *t* = −3.67, *p* = 0.124), when the two mediators, FLE and FLA, were controlled. This indicates that FLE and FLA jointly played the role of full mediation between grit and FLP. Grit increased FLP, mainly by increasing levels of FLE and decreasing levels of FLA.

**Table 5:** Regression analysis results: FLE and FLA as mediators between grit and FLP.

**Regression equations**		**Fit summary**			**Coefficient**		
**Predictor**	**Outcome**	***R***	***R*^**2**^**	***F***	**β**	***B***	***t***
Grit	FLP	0.15	0.02	5.13^***^	0.13	4.20	3.39^***^
Grit	FLE	0.45	0.20	59.28^***^	0.46	0.47	32.20^***^
Grit	FLA	0.43	0.19	51.95^***^	−0.42	−0.49	−29.40^***^
Grit	FLP	0.37	0.14	21.43^***^	−0.06	−2.12	−3.67
FLA					−0.30	−8.56	−16.92^***^
FLE					0.14	4.58	7.96^***^

*FLE, Foreign Language Enjoyment; FLA, Foreign Language Anxiety; β, standardized coefficient; B, unstandardized coefficient*;

*** *p < 0.001*.

To test the validity of the finding that FLE and FLA mediate between grit and FLP, a bootstrap sampling method using 5,000 iterations was employed. As Table 6 illustrates, the 95% confidence interval of total indirect effect for both of the mediators was [0.142, 0.240], which did not include 0. This indicates that when tested simultaneously, FLE and FLA significantly mediated the relationship between grit and FLP.

**Table 6:** Analysis of mediating effects of FLE and FLA between grit and FLP.

**Effect**	**Mediators**	**Point estimate**	***SE***	***BC*a 95% *CI***
Indirect effects	FLE	0.06	0.020	0.028, 0.105
	FLA	0.13	0.022	0.084, 0.170
	Total	0.19	0.025	0.142, 0.240
Contrast	FLA/FLE	0.06	0.033	0.126, 0.009

*FLE, Foreign Language Enjoyment; FLA, Foreign Language Anxiety; FLP, Foreign Language Performance*.

Similarly, the 95% confidence intervals of the effect sizes of FLE and FLA were [0.028, 0.105] and [0.084, 0.170], respectively, and neither included 0, suggesting that when tested separately with the other controlled, FLA and FLE significantly mediated the effect of grit on FLP. Further examination of the contrast between the mediating effects of FLA and FLE showed that the 95% confidence interval did not include 0, indicating that the mediating effect of FLA was significantly stronger than that of FLE on the relationship between grit and FLP.

## Discussion

### Tendency in Students' Levels of Grit, FLE, and FLA

The first research question sought to explore the general profile of students' grit, FLE, and FLA. We found a general tendency for students to exhibit moderate-high levels of grit, moderate-high levels of FLE, and low-moderate levels of FLA.

More than half of the students perceived themselves as having a moderate-high level of grit. The mean score of grit among the high school students in this study was lower than that among middle school students reported in the study conducted by Wei et al. (2019). Although beyond the assumption that grit increases with age (Duckworth et al., 2007), this result echoed the findings of Credé et al. (2017) meta-analysis, which reported an inconsistent effect of demographic factors, including age, on grit level. This inconsistency may have resulted from the narrow age range of the participants (i.e., 14–16) and the homogeneity of the instructional level (i.e., first-year high school students).

More than half of the students in this study experienced a moderate-high level of enjoyment and a low-moderate level of anxiety. The mean score of enjoyment among first-year high school students in this study was higher than that among second-year high school students as reported in Li (2018), but the mean score of anxiety was lower. This gap may have emerged as a result of the discrepancy between the participants' academic levels; the first-year high school students surveyed in the current study were under less academic pressure than the second-year high schoolers in Li (2018). Because the National College Entrance Examination is highly competitive (Davey et al., 2007), it can put more pressure on second-year high school students and hence make them more likely to be overwhelmed by negative feelings than first-year high school students.

The mean scores of students' enjoyment and anxiety in this study were found to be lower than those of the students in the undergraduate sample surveyed in Jiang and Dewaele (2019). Undergraduate school students' tendency to learn skills rather than study to pass exams may account for such a difference. For most high school students, the high-stakes National College Entrance Examination has a washback effect on teaching and learning. This stimulates a more test-oriented mode of teaching (Kirkpatrick and Zang, 2011), which can make language learning tedious and therefore a major source of negative feelings. This academic environment stands in sharp contrast to that of English major students in college who no longer have test-driven classes but still face the fierce competitiveness of the job market.

When compared with the international sample in Dewaele and MacIntyre (2014), the participants in this study had lower mean scores for both enjoyment and anxiety. These results are in line with the findings of MacIntyre et al. (2019a), who found that Chinese students experienced significantly lower levels of positive emotion than students in the international sample (but did not find that Chinese students experienced more negative emotions). According to Dewaele et al. (2018), factors such as individual, teacher, and contextual variables can make a difference in these analyses. China has the largest number of foreign language learners in the world (You and Dörnyei, 2016), and most students are required to learn a foreign language for years before going to university. Therefore, trying to promote the importance of the emotional experience of language learning is of vital importance for improving the well-being of foreign language learners in China. There is a considerable range of options available to help students to experience fewer negative emotions and foster positive emotions in language learning.

### Interconnectedness of Grit, FLE, FLA, and FLP

The second question sought to examine the correlation between grit, FLE, FLA, and FLP. Significant correlations were found between all of these variables. Grit was found to be positively and moderately correlated with FLE, which echoes the findings of previous research that grit is consistently linked with positive emotions (Hill et al., 2016; Wei et al., 2019; Lee, 2020). Furthermore, the results showed that grit was negatively and moderately correlated with FLA, dovetailing with the findings of Datu et al. (2019), who reported a negative correlation between grit and depression. This indicates that gritty individuals tend to experience more positive emotions and fewer negative emotions.

Grit was found to be significantly correlated with FLP, positively but weakly, which is consistent with the findings of Wei et al. (2019). The weak correlation between grit and FLP suggests that FLP can be mediated or moderated by other variables, such as classroom environment and emotions, among many others (Credé et al., 2017). It seems that gritty individuals have not only higher levels of enjoyment and lower levels of anxiety but also higher levels of language performance. It is likely that gritty individuals, when faced with challenges and adversity, tend to maintain motivation and focus more on their long-term goals rather than on their negative emotions. The achievement resulting from motivation and grit will, in turn, lead them to experience more enjoyment in the pursuit of their long-term goals (Datu et al., 2019). The findings empirically support the broaden-and-build theory that positive emotions (i.e., enjoyment) can broaden personal resources (i.e., FLP), whereas negative emotions (i.e., anxiety) may narrow them (Fredrickson, 2001; MacIntyre and Gregersen, 2012b).

We found that FLE was negatively correlated with FLA, which dovetails with the findings of previous studies (Dewaele and MacIntyre, 2014; Dewaele et al., 2018; Jiang and Dewaele, 2019). Furthermore, this result confirms the claim that FLE and FLA are not at “opposite ends of a dimension,” which means that it is possible that individuals may experience both high levels of FLE and FLA and low levels of FLE and FLA (Dewaele and MacIntyre, 2014; MacIntyre et al., 2019a). FLP was also found to positively correlate with FLE and negatively correlate with FLA, and the strength of the correlation between FLP and FLA was stronger than that between FLP and FLE, which is in line with the findings of Li (2018), but different from the findings of Dewaele and MacIntyre (2014). This result confirms that emotions are important and influential factors of language learning (MacIntyre et al., 2019a). It also reveals that the potential relative effect of positive and negative emotions may differ across cultural contexts.

Furthermore, the findings revealed that negative emotions may have more influence on language performance than positive emotions in the Chinese context. Chinese students, especially high school students, are under huge academic pressure (Li, 2017), which is inevitably a source of negative emotions. The additional negative emotions caused by academic pressure may exacerbate the negative emotional experience in the language learning classroom, which in turn affects language performance more than positive emotions would. However, the moderate-high correlation of enjoyment and anxiety and the low-medium correlation between enjoyment and language performance indicate that positive emotions are potential factors of the alleviation of negative emotions (Fredrickson, 2013), therefore increasing language performance.

### FLE and FLA as Mediators

The third research question sought to explore the mediating effect of FLE and FLA on grit and FLP. It was found that the two emotions played a full mediation role, meaning that grit was associated with FLP mainly through the two emotions (i.e., FLE and FLA). This may explain the inconsistent findings of grit as a predictor of academic success in a meta-analysis conducted by Credé et al. (2017). The result also confirms the claim that grit is a crucial factor of language learning that requires persistent effort of second language learners (MacIntyre et al., 2019b). Chinese students are under great pressure because they are externally motivated to meet their parents' expectations or simply pass their exams, rather than being intrinsically motivated to integrate themselves into the target culture (You and Dörnyei, 2016; MacIntyre et al., 2019a). In this case, grit seems to play a key role in students' language performance, especially for students with low levels of proficiency.

Mediation analysis also revealed that both positive and negative emotions (i.e., FLE and FLA) can mediate the relationship between grit and FLP. Furthermore, the mediating effect of FLA is stronger than that of FLE. The results indicate that anxiety may still be the most relevant emotion to language learning performance in the Chinese high school context. The results also suggest that grit improves FLP by increasing enjoyment and decreasing anxiety. These results further highlight the importance of emotions in improving FLP, because emotions may influence attitudes toward and motivation for language learning (MacIntyre et al., 2019a). Foreign language learning is required at all levels of education in China, and FLP is an important criterion for college admissions and job requirements. The stronger mediating effect of anxiety as opposed to that of enjoyment further indicates that due attention should be paid to students' emotions, especially anxiety in the Chinese context, to improve students' well-being and FLP (Li, 2018).

## Conclusion and Implications

This study explores the relationships between grit, FLE, FLA, and FLP and the possible mediating effects of FLE and FLA between grit and FLP. It reveals that most of the students surveyed had moderate-high levels of grit and FLE and low-moderate levels of FLA. Furthermore, the three variables were in close relationship with FLP. While both positive and negative emotions played full mediating roles between grit and FLP, the mediating effect of the negative emotion of FLA was stronger than that of the positive emotion of FLE.

These findings suggest that teachers need to foster students' grit levels for the reason that increased grit levels may improve FLP and positive emotional experience and also have the potential effect of alleviating negative emotions. For example, teachers could introduce celebrities who are both gritty and successful as examples for students to follow. Enhancing students' feelings of having an indomitable spirit might enable them to overcome difficulties in language learning.

Furthermore, teachers are advised to be more aware of students' emotional states. Because the two emotions of enjoyment and anxiety may play key roles in FLP, students' experiences of enjoyment should be augmented, which in turn may reduce levels of anxiety. Teachers themselves should be positive and maintain a good mood to create a positive and supportive classroom environment in which students with positive peers can make the most of language learning classrooms and improve their language learning performance.

Finally, teachers should reduce instances of anxiety-provoking situations as much as possible, because the effects of negative emotions are usually more obtrusive than the effects of positive emotions. For example, organizing group work instead of individual work may encourage students' cooperative learning and lessen feelings of competition. Given that enjoyment potentially alleviates anxiety, creating a harmonious and supportive classroom environment would produce a desirable scaffolding effect and thus reduce students' anxiety.

## Limitations and Future Research Suggestions

Despite these important findings on the relationships among grit, FLE, FLA, and FLP, this study is limited in two ways.

First, the convenience sampling method used in this study has a deficit of homogeneity in age, instructional level, cultural background, and language context, limiting the generalizability of its findings. Future studies could overcome this deficit by observing a larger sample of students from different age groups, social backgrounds, and geographical areas.

Second, the cross-sectional method adopted in this study is helpful for a general understanding of how grit and emotions interact and relate to FLP, but does not allow the investigation of changes over time in students' emotional status and the interacting effects of their grit levels and FLP. Therefore, a longitudinal design is suggested for future studies to yield profound findings regarding these factors.

## Data Availability Statement

The raw data supporting the conclusions of this article will be made available by the authors, without undue reservation.

## Ethics Statement

An ethical review and approval were not required for this study on human participants in accordance with local legislation and institutional requirements. Written informed consent to participate in this study was provided by the participants and their legal guardians/next of kin.

## Author Contributions

EL collected and analyzed the data and wrote the first draft of the manuscript. EL and JW reviewed and revised the manuscript. All authors contributed to the article and approved the submitted article.

## Conflict of Interest

The authors declare that the research was conducted in the absence of any commercial or financial relationships that could be construed as a potential conflict of interest.
